# Isolated steroid-resistant nephrotic syndrome in a Chinese child carrying a de novo mutation in WT1 gene:a case report and literature review

**DOI:** 10.1186/s12887-022-03358-3

**Published:** 2022-06-16

**Authors:** Yiyang Li, Chuan Tian, Yajun Wang, Guoda Ma, Riling Chen

**Affiliations:** 1 Department of Pediatrics, Affiliated Hospital of Guangdong Medical University, Zhanjiang City, Guangdong Province China; 2 Department of Pediatrics, Shunde Women and Children’s Hospital of Guangdong Medical University (Maternity and Child Healthcare Hospital of Shunde Foshan), Foshan, Guangdong Province China

**Keywords:** Isolated Steroid-resistant Nephrotic syndrome, WT1 gene

## Abstract

**Background:**

Isolated steroid-resistant nephrotic syndrome (ISRNS) is caused by mutations in the Wilms’ tumor-1 (WT1) gene, which encodes glomerular podocytes and podocyte slit diaphragm.We report a novel 8-year-old female patient with ISRNS carrying a de novo missense mutation in WT1 gene and presenting a new type of pathology, have never been reported.We also systematically review previous reports of ISRNS in Chinese children.

**Case presentation:**

A 8-year-old Chinese patient who had steroid-resistant nephrotic syndrome,responded poorly to immunosuppressant, and had no extrarenal manifestations. The patient had a female phenotype and karyotype of 46, XX. A new type of renal pathology, proliferative sclerosing glomerulonephritis (PSG),and a de novo missense mutation in WT1 gene, c.748C > T (p.R250W),which have not yet been reported, were identified. She was diagnosed with ISRNS.The patient progressed to end-stage renal disease at the age of 10 years,underwent dialysis and kidney transplant. Renal function and urine protein were normal during 4-year follow-up.

**Conclusions:**

WT1 gene testing should be performed to guide treatment for patients with steroid-resistant nephrotic syndrome, especially for isolated cases and female patients.

## Background

Isolated steroid-resistant nephrotic syndrome (ISRNS) is caused by mutations in the Wilms’ tumor-1 (WT1) gene (OMIM 607,102), which encodes glomerular podocytes and podocyte slit diaphragm [1–3]. The WT1 gene mutation is found in 6% to 7% of patients with ISRNS younger than 18 years of age and in 10% to 12% of female patients [4, 5]. The age of onset ranges from birth to adolescence [1–3]. It responds poorly to immunosuppressants and has no extrarenal manifestations, such as Wilms’ tumor or urogenital malformations [1]. The main types of renal pathology are diffuse mesangial sclerosis (DMS) and focal segmental glomerulosclerosis (FSGS) [3, 6]. It is progressively worsening and progresses to end-stage renal disease (ESRD) 0.1 to 11 years after the onset of disease [3, 7]. We report a case of an 11-year-old female child with ISRNS caused by de novo mutation in the WT1 gene, presenting a new type of pathology. We also systematically review previous reports of ISRNS in Chinese children and summarize experience and progress in its diagnosis and treatment.

## Case presentation

The patient was female, and experienced foamy urine and symmetrical edema of lower limbs at the age of 8 years. Physical examination: 26 kg, blood pressure 130/100 mmHg(90–110/60-75 mmHg), moon-shaped face, buffalo back, hairy upper arms, no obvious swelling of eyelids or lower limbs, no palpable mass in the abdomen, and normal external genitalia. There were no abnormalities in visual, hearing, and intelligence tests.

**Fig. 1:**
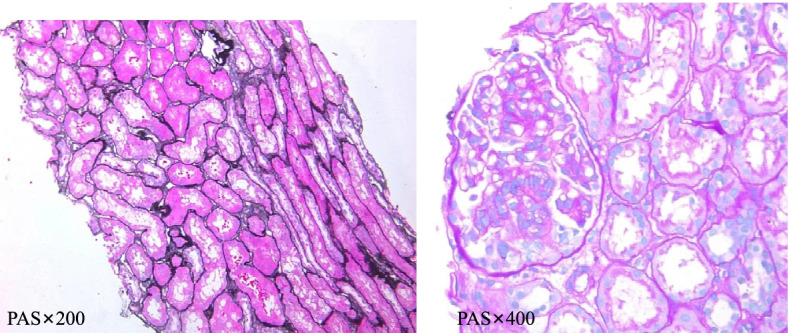
Renal pathology of the proband Pathological findings on periodic acid-Schiff (PAS) staining: mild focal segmental mesangial cell proliferation and mesangial matrix expansion, no proliferation of endothelial cells, no thickened basement membrane, patchy atrophy of tubules, vacuolar and granular degeneration and shedding of tubule epithelial cells, shedding of brush border, visible protein casts, red blood cells visible in the lumen of focal tubules; diffuse interstitial edema with infiltration of mainly monocytes/lymphocytes, multifocal fibrosis; and endothelial thickening of arterioles. Immunofluorescence findings: C3 deposition in the mesangial area and capillary wall; and negative for IgG, IgA, IgM, C1q, Fib, HBsAg, and HBcAg

Urinalysis showed urine protein 3 + (226 mg/kg.d). Blood chemistry revealed albumin 28.7 g/L(38-54 g/L), urea nitrogen 10.48 mmol/L(2.48–8.07 mmol/L), creatinine 161.3 umol/L(34.0–80.0umol/L), cholesterol 13 mmol/L(3.1–5.7 mmol/L), glomerular filtration rate (GFR) 38.79 ml/min*1.73 m^2^ (> 90 ml/min*1.73 m^2^);mild anemia, normal complement C3 levels, negative anti-streptolysin O, negative hepatitis B virus antigen; and no abnormalities in autoantibodies (antinuclear antibodies, anti-dsDNA), T cell subsets, immunoglobulins, and erythrocyte sedimentation rate. Color ultrasound and computed tomography (CT) of the genitourinary system showed no tumors or developmental abnormalities. The renal pathology suggested proliferative sclerosing glomerulonephritis (PSG) (Fig. 1).

The patient had no history of tumor, hepatitis, allergic purpura, medication taken without renal impairment. Birth history was uneventful. There was no consanguinity and birth history was uneventful. There was no family renal disease.

The diagnosis was nephritic syndrome, chronic renal failure (stage III), renal hypertension, and renal anemia.The patient was treated with initial 2 mg/kg.d oral prednisone.Urine protein remained positive during the treatment for more than 3 months, fluctuating between 2 + and 3 + .After the type of pathology was identified, 25 mg/kg.d of oral mycophenolate mofetil (MPA) was also prescribed, combined with benazepril and erythropoietin to manage her hypertension and anemia, respectively, and dietary intervention was also carried out. A follow-up evaluation 6 months later showed morning urine protein remaining 3 + and areduction in 24-h urine protein (42 mg/kg.d). Blood chemistry suggested hypercholesterolemia, azotemia, and normal albumin levels. Patient didn’t achieve remission. GFR was 37.36 ml/min*1.73 m^2^, indicating no significant progress in chronic renal failure. Hence, MPA was changed to cyclophosphamide (CTX), which was administered every 2 weeks for a total of five treatment cycles to make a cumulative dose of 104 mg/kg, but no remission was achieved.

She progressed to ESRD at the age of 10 years and underwent hemodialysis for 9 months. Kidney transplantation was performed at the age of 10 years and 9 months. The patient was followed up regularly. The renal function and urine protein were normal during 4 years of follow-up.

### Karyotype and genetic testing

**Fig. 2:**
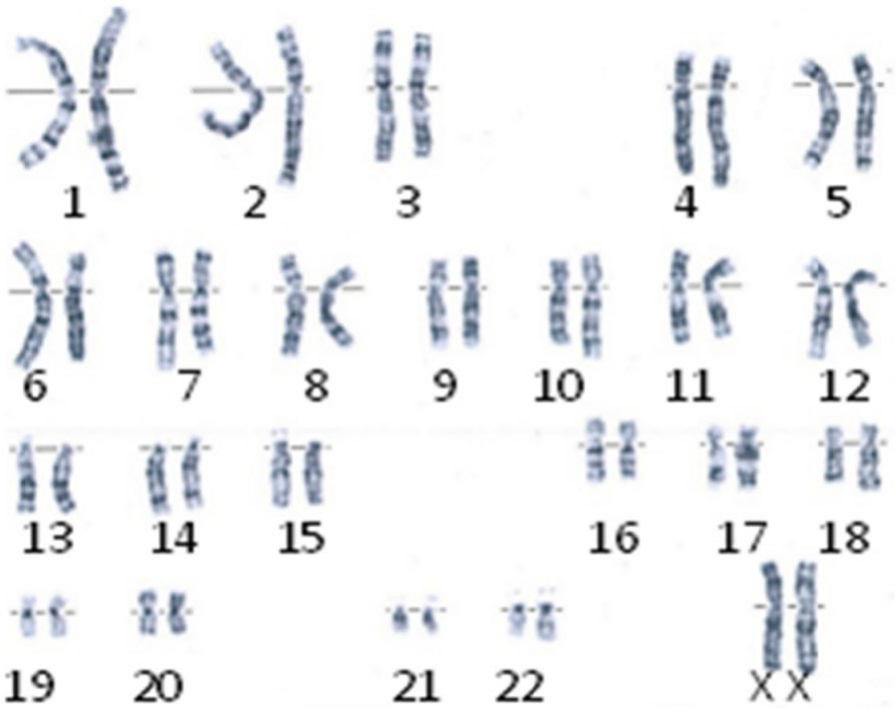
Karyotype: 46, XX

In order to confirm the diagnosis, a blood sample was collected from the patient after informed consent was obtained from her parents. Chromosome karyotype was 46, XX (Fig. 2). Hereditary nephrotic syndrome-related genes were sequenced. (Table 1) After suspected pathogenic variants were detected, peripheral blood was collected from the patient’s parents and younger brother for pedigree verification by Sanger sequencing. It was found that the proband had a heterozygous mutation in exon 9 of the WT1 gene on chromosome chr11:32,413,566, that is, c.748C > T. Specifically, nucleotide 748 in the coding region was changed from cytosine to thymine, which caused amino acid 250 to be changed from arginine to tryptophan, that is, p.R250W, which was a missense mutation. The transcript was NM_001198551.

**Table 1:** Analytical genes associated with hereditary nephrotic syndrome list

Number	Symbol	Number	Symbol	Number	Symbol	Number	Symbol	Number	Symbol
1	ACTN4	16	THSD7A	31	PLA2R1	46	CRB2	61	LAMB3
2	ADCK3	17	TRPC6	32	PLCE1	47	CUBN	62	CD2AP
3	ADCK4	18	TSC2	33	PMM2	48	DGKE	63	KANK1
4	ALG1	19	UMOD	34	PTPRO	49	EMP2	64	KANK2
5	ANLN	20	WDR73	35	SCARB2	50	NPHP1	65	KANK4
6	APOA1	21	WT1	36	MYH9	51	CFH	66	LAMA3
7	APOE	22	XPO5	37	GLA	52	COL4A3	67	LAMB2
8	APOL1	23	LYZ	38	INF2	53	COL4A4	68	MME
9	ARHGAP24	24	ARHGDIA	39	NPHS1	54	COL4A5	69	ZMPSTE24
10	COQ6	25	B2M	40	NPHS2	55	COQ2	70	LAMC2
11	MYO1E	26	CD151	41	NUP107	56	MEFV	71	LMX1B
12	NEIL1	27	PAX2	42	NUP205	57	FAT1	72	COL4A6
13	NEK8	28	PDSS1	43	NUP93	58	FGA	73	ITGA3
14	SLC35A2	29	PDSS2	44	SLC17A5	59	FLG	74	ITGB4
15	SMARCAL1	30	PEX1	45	COQ9	60	FN1		

This mutation is not present in the 1000 Genomes Project or Exome Aggregation Consortium database. Pathogenic variants of this mutation, which have the same amino acid change, are registered in the Human Gene Mutation Database (HGMD) (ID CM107177 and ID CM910411). However, there is no report about the heterozygous mutation c.748C > T causing ISRNS. This is a new mutation site. This mutation is not a polymorphic locus and occurs at an extremely low frequency. Mutation Taster predicts that this mutation can change the splice site. PhyloP and PhastCons Nucleotide conservation scores were 2.917 and 1, respectively. The sequence of amino acid at the missense mutation site was aligned with homologous sequences of other species. The results are shown in Table 2.The amino acid at this site of WT1 is highly conserved among human, chimpanzee, rhesus, mouse, chook, Fugu rubripes, zebra fish, fruit fly, nematode, and African melon toad. This variant was classified as a pathogenic variant in HGMD, ClinVar, and Mutation Taster.

**Table 2:** Homology comparison of WT1 amino acid sites corresponding to missense mutations

Human (Homo sapiens)	250	FQCKTCQRKFS	**R**	SDHLKTHTRTHT
Chimpanzee (Ptroglodytes)	325	TCQRKFS	**R**	SDHLKTHTRTH
Rhesus (Mmulatta)	462	TCQRKFS	**R**	SDHLKTHTRTH
Mouse (Mmusculus)	462	TCQRKFS	**R**	SDHLKTHTRTH
Chook (Ggallus)	362	CKTCQRKFS	**R**	SDHLKTHTRTH
*Fugu rubripes* (Trubripes)	361	CETCQRRFA	**R**	SDHLKTHTRTH
zebra fish (Drerio)	364	YTCKVCGQVFS	R	SDHLSTHQRTH
Fruit fly (Dmelanogaster)	665	YTCKVCGQVFS	**R**	SDHLSTHQRTH
Nematode (Celegans)	165	FQCRTCLRSFS	**R**	**S**DHLAKHERTH
African melon toad (Xtropicalis)	368	FQCKTCQRKFS	**R**	SDHLKTHTRTH

**Fig. 3:**
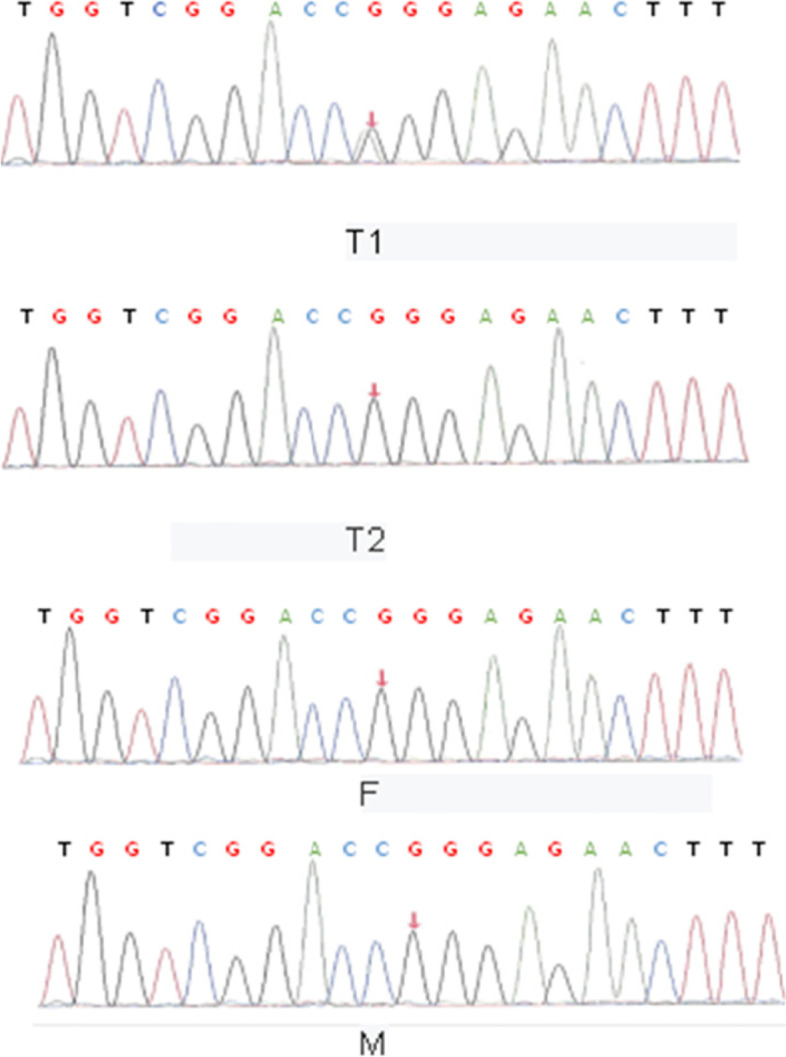
Sequences of the WT1 gene mutation of the proband and her younger brother and parents. T1 (II2) proband; T2 (II1) proband’s younger brother; F (I1) proband’s father; M (I2) proband’s mother

The pedigree analysis showed that no mutation at this site was found in the proband’s parents or younger brother with a normal clinical phenotype. This mutation was co-segregated with the disease in the family (Fig. 3).

According to the 2015 American College of Medical Genetics and Genomics (ACMG) guideline, this variant is a pathogenic variant (PS1 + PS2 + PS4 + PM1 + PM2 + PM5 + PP1 + PP3).

## Discussion

The WT1 gene is located on chromosome 11p13 and contains 10 exons. WT1 is a zinc finger-like transcription factor. The amino terminus is rich in proline and glutamic acid, is encoded by exons 1 to 6, and can activate transcription. The carboxyl terminus contains 4 zinc finger domains that can bind to DNA, each consisting of 2 cysteines and 2 histidines, and are encoded by exons 7 to 10, respectively [3, 4, 8]. The two subtypes, WT1 + KTS and WT1-KTS, produced by the insertion of a tripeptide amino acid fragment composed of lysine-threonine-serine (KTS) encoded by exon 9 between the 3rd and 4th zinc fingers have clear functions [3, 4, 8]. WT1 + KTS plays an important role in maintaining the normal function of podocytes. WT1-KTS is essential for the development of embryonic kidney and gonads. An appropriate WT1 + KTS/-KTS ratio (the normal ratio is close to 2:1) is indispensable for the normal development of the kidney and urogenital system [3, 4, 8].

In this study, the heterozygous mutation in exon 9 of the WT1 gene, c.748C > T, resulted in the mutation of amino acid 250 from arginine to tryptophan. On the one hand, the WT1 gene mutation produces an allele that only expresses the -KTS subtype, resulting in an abnormal WT1 + KTS/-KTS ratio, which can lead to abnormal kidney development [8]. On the other hand, as a nuclear transcription factor, WT1 can bind to the promoters and enhancers of 18 podocyte disease-related mutant genes, such as Nphs1, Nphs2, Actn4, and CD2AP [8, 9]. Mutations in exon 9 affect the binding of the zinc finger domains to DNA and affect the recognition and binding of WT1 to target genes, thereby affecting target gene transcription. Moreover, these target genes have been proven to be down-regulated when WT1 is absent, resulting in abnormal expression of protein molecules, such as nephrin, podocin, a-actinin 4, and CD2AP, that maintain the stable structure of the podocyte slit diaphragm, which impairs the stability of podocyte actin, thereby being involved in the development of glomerulosclerosis. Various mutations in the WT1 gene have been identified as the causes of hereditary FSGS and DMS [7–9], which eventually lead to the loss or functional changes of the slit diaphragm, resulting in damage to the filtration barrier and causing pathological proteinuria.

A literature search was conducted in China National Knowledge Internet (CNKI), Wanfang Database, and PubMed with “WT1 gene”, “Chinese”, “children”, “isolated”, “steroid-resistant”, and “nephrotic syndrome” as keywords [2, 4, 10, 11]. As shown in Table 3, a total of16 cases of ISRNS were retrieved. Among them, 15 patients were female and only 1 was male, suggesting that this disease is more likely to occur in female children. The average age of onset was 3.4 years. The earliest onset was at birth. It progressed to ESRD in an average of 1.1 years, and even started with acute renal failure. Regarding the type of pathology, there were 5 cases of DMS, 4 cases of FSGS, 1 case of MCD. The type of pathology, PSG, described in this paper has not been reported before. The WT1 gene mutations that cause ISRNS are mainly heterozygous missense mutations in exons 8 and 9 and splicing mutations in intron 9. The most common mutation is c.1180C > T. The mutation site described in this paper has not been reported before.

**Table 3:** literature reports on ISRNS caused by WT1 gene mutation in Chinese children

References		gender	The onset age(year)	Age of ESRD Onset(year)	Mutations Region	Mutations type	Sequence Changes	Protein Changes	Renal Pathology	Therapy	treatment response	Renal outcome
1	This report	female	8	10	Exon 9	Missense mutation	c.748C > T	p.R250W	PSG	MP → P + MMF → CTX → HD → KT	Resistant	ESRD
2	Li J,Ding J, et al. [12]	female	0.3	3	Exon 9	splice mutation	c.IVS9 + 5G > A	p.D396N	FSGS	GC → CsA	Resistant	ESRD
3		female	1	1.3	Exon 9	Splice mutation	c.IVS9 + 5G > A	p.D396N	—	GC → CTX	Resistant	ESRD
4	Liang-zhong sun.et al. [2]	female	0.5	0.5	Exon 8	Missense mutation	c.1097G > A	p.R366H	DMS	_	—	_
5		female	—	—	Exon 8	Missense mutation	c.1097G > A	p.R366H	—	_	—	_
6		female	—	—	Exon 9	Missense mutation	c.1180C > T	p.R394W	FSGS	P → FK506	Complete remission	Normal
7		female	0.1	0.1	Exon 9	Missense mutation	c.1180C > T	p.R394W	DMS	_	—	_
8		female	—	9	Exon 9	Missense mutation	c.1180C > T	p.R394W	DMS	P → HD → KT	Resistant	Normal
9	Yue Z, et al [4]	female	0.4	0.4	Intron 9	splice mutation	c.1228 + 4C > T	_	_	-		ESRD
10		female	9	_	Intron 9	splice mutation	c.1228 + 4C > T	_	FSGS	P + MP → MZ → MMF → FK506	—	Normal
11		female	1	6.8	Intron 9	splice mutation	c.1228 + 5G > A	_	MCD	P → MMF → MP + CSA → FK506	improve	ESRD
12		female	5	_	Exon 9	Missense mutation	c.1180 C > T	p.R394W	FSGS	P → FK506	Resistant	Normal
13		female	0	0.2	Exon 9	Missense mutation	c.1180 C > T	p.R394W	DMS	-	Complete remission	ESRD
14	Yang Yonghui et al. [11]	female	0.5	0.5	Exon 8	Missense mutation	c.1097 G > A	p.R366H	DMS	-	—	ESRD
15		female	2	_	Exon 9	Missense mutation	c.1180C > T	p.R394W	_	-	—	Normal
16		female	8.1	_	Exon 9	splice mutation	c.1051A > G	p.D396N	_	_	Resistant	
17		male	6.3	6.3	Exon 8	Missense mutation	c.1051A > G	p.K351E	_	HD	Resistant	ESRD

*CSA* Cyclosporine A, *CTX* Cyclophosphoramide, *DMS* Diffuse Mesangial Sclerosis, *ESRD* End-stage Renal Disease, *FK506* tacrolimus, *FSGS* Focal Segmental Glomerular Sclerosis, *GC* Glucocorticoid, *HD* Hemodialysis, *PSG* Proliferative Sclerosing Glomerulonephritis, *KT* Kidney Transplantation, *MCD* Minimal Change Disease, *MMF* Mycophenolate Mofetil, *MP* Methylprednisolone, *NC* Not Clear, *P* Prednisone

ISRNS is resistant to glucocorticoids and responds poorly to most immunosuppressants. Hence, it is recommended not to use glucocorticoids and to use immunosuppressants with caution [1, 4, 12]. At present, the treatment is mainly to reduce proteinuria, protect kidney function, and delay disease progression. It can quickly progress to ESRD. In this case, the best treatment is kidney transplantation, because of the low recurrence rate [13]. Sun et al. reported that 3 children with FSGS were treated with tacrolimus (FK506), which induced partial response in 1 patient and complete response in patients [2]. It has also been reported that cyclosporine (CsA) effectively reduced urine protein in hereditary nephrotic syndrome caused by WT1 gene mutations, and therefore, calcineurin inhibitors (FK506 and CsA) have a certain effect in the treatment of hereditary nephrotic syndrome caused by WT1 gene mutations, which may be achieved by stabilizing the podocyte actin cytoskeleton [1, 12, 14, 15]. However, the efficacy needs to be further confirmed by a multi-center, large-scale randomized controlled study.

In addition to causing ISRNS, WT1 gene mutations can also result in Denys-Drash syndrome (DDS), Frasier syndrome (FS), WAGRS, which are often accompanied by male pseudohermaphroditism, hypospadias and other urological malformations, gonadal tumors, Wilms’ tumor, and other manifestations [1, 3, 16]. DDS, FS, and WAGRS are easier to identify due to extrarenal manifestations. Therefore, for children with a female phenotype and ISRNS with DMS or FSGS, detailed physical and imaging examinations (e.g., B-ultrasound and CT) of the genitourinary system should be performed to exclude genitourinary malformations, tumors, and other lesions, and chromosome karyotype and WT1 gene mutation analysis should be performed to identify patients with ISRNS [16] to avoid unnecessary treatment with steroids and other immunosuppressants. Children with ISRNS caused by WT1 gene mutations progress to ESRD 0.1 to 11 years after the onset, which is not only about 10 years faster than those without gene mutations, but also than faster those with hereditary nephrotic syndrome caused by mutations in other genes (e.g., NPHS2, NPHS1, PTPRO, and LAMB1) [17]. Therefore, WT1 mutation analysis is also helpful for prognosis evaluation. Although most of the WT1 gene mutations were new [2, 4, 7], there are also reports of mothers passing the mutated gene to their children [7, 10, 18]. Therefore, WT1 mutation analysis can be used in genetic counseling and prenatal genetic diagnosis for families at high risk of WT1 mutations.

The clinical manifestations of the patient reported in this study were ISRNS,with the new renal pathology type, PSG and de novo mutation in the WT1 gene, c.748C > T. Pelletier et al. reported that mutations at this site could cause DDS [19]. Therefore, it is necessary to follow up this patient to detect possible gonadal tumors and Wilms’ tumors, etc.

In conclusion,WT1 gene testing should be performed to guide treatment for patients with steroid-resistant nephrotic syndrome, especially for isolated cases and female patients.

## Data Availability

The datasets generated and analysed during the current study are available in the https://www.uniprot.org/uniprot/P19544 repository”.

## Abbreviations

ISRNS: Isolated Steroid-resistant Nephrotic Syndrome
WT1: Wilms’ tumor-1 gene
PSG: Proliferative Sclerosing Glomerulonephritis
DMS: Diffuse Mesangial Sclerosis
FSGS: Focal Segmental Glomerulosclerosis
ESRD: End-stage Renal Disease
GRF: Glomerular Filtration Rate
CT: Computed Tomography
PSG: Proliferative Sclerosing Glomerulonephritis
MPA: Mycophenolate
CTX: Cyclophosphamide
HGMD: Human Gene Mutation Database
ACMG: American College of Medical Genetics and Genomics
FK506: Tacrolimus
CsA: Cyclosporine
DDS: Denys-Drash syndrome
FS: Frasier syndrome.GC:glucocorticoid
HD: Hemodialysis
PSG: Proliferative sclerosing glomerulonephritis
KT: Kidney transplantation
MCD: Minimal change disease
MMF: Mycophenolate Mofetil
MP: Methylprednisolone
P: Prednisone
